# Motor Vehicle Collision Patient with Simultaneous Duodenal Transection and Thoracic Aorta Injury: A Case Report and Review of the Literature

**DOI:** 10.1155/2015/519836

**Published:** 2015-01-26

**Authors:** Charlie Chen, Kevin Schuster, Bishwajit Bhattacharya

**Affiliations:** Surgical Critical Care and Surgical Emergencies, Section of Trauma, Yale School of Medicine, 330 Cedar Street, BB310, P.O. Box 208062, New Haven, CT 06520-8062, USA

## Abstract

Blunt polytrauma can present complex management decisions. Here we report the case of a 31-year-old male involved in a high speed motor vehicle collision resulting in both duodenal and thoracic aorta injury that was managed collaboratively between the trauma, vascular, and cardiothoracic surgical teams. The patient went on to a full recovery. We also review the management of such injuries which has evolved over the past two decades resulting in less morbidity and mortality.

## 1. Introduction

Duodenal traumas constitute less than 5% of all abdominal traumas [1] and are usually penetrating. Blunt thoracic aorta injuries occur in less than 1% of motor vehicle accidents, but are responsible for 16% of deaths [2]. In this case we report the management of a simultaneous blunt duodenal and aorta injury that resulted in a good outcome.

## 2. Case

**Figure 1:**
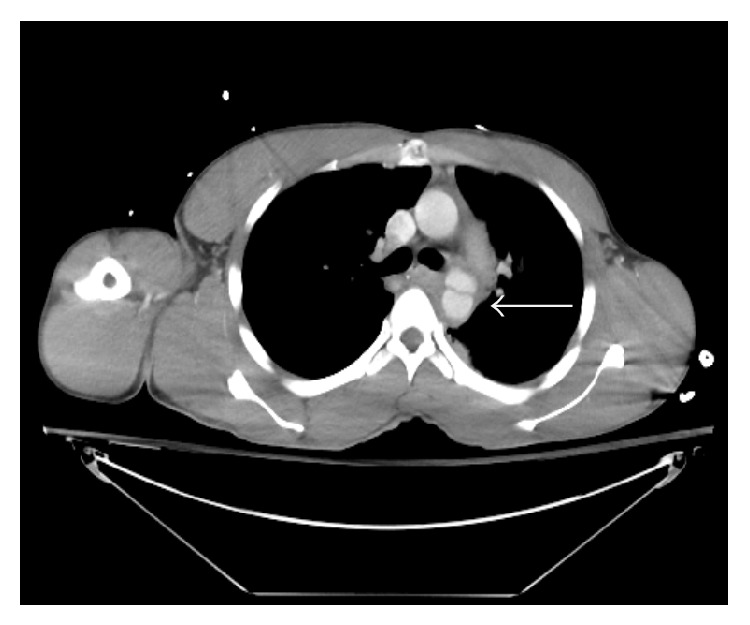
Aortic injury with pseudoaneurysm noted in the region of the ligamentum arteriosum. Hematoma is also noted surrounding the distal aortic arch and the descending thoracic aorta.

**Figure 2:**
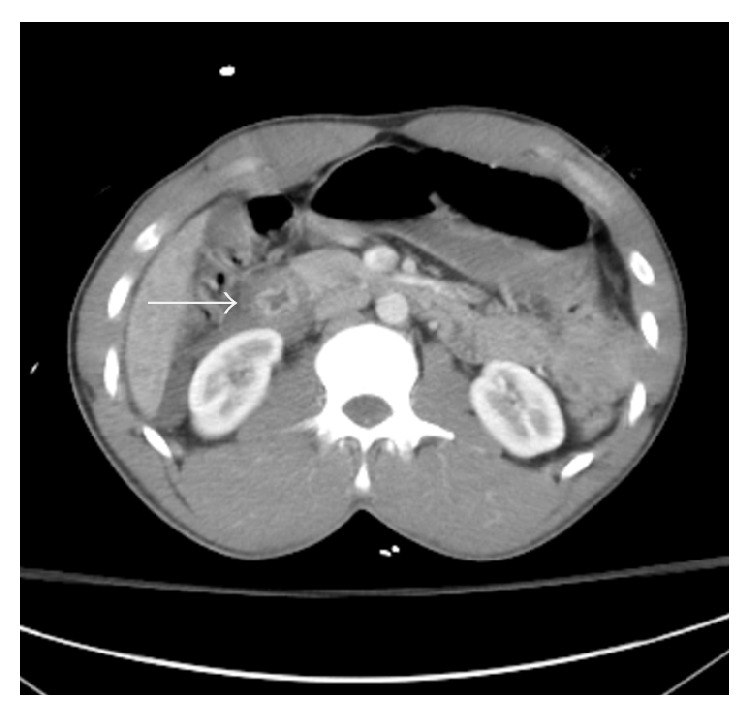
Discontinuity of the wall of the second portion of the duodenum is noted with surrounding intermediate attenuation fluid. Multiple foci of intra-abdominal free air are also present.

A 31-year-old intoxicated, unrestrained male was brought in by EMS after crashing his car into a truck. On arrival he was hemodynamically appropriate. He complained of right arm and abdominal pain. Primary survey revealed no deficits. The secondary survey revealed tenderness over the anterior chest wall and the right upper quadrant of the abdomen. His right upper extremity had an obvious deformity with motion limited by pain but with no vascular compromise. A chest X-ray was obtained in the trauma bay which only demonstrated a single right side fourth rib fracture. His initial hemoglobin was 14.3 g/dL. The patient's physical exam was complicated by alcohol intoxication and therefore underwent CT imaging of his brain, chest, abdomen, pelvis, and spine column to rule out life threatening injuries. Imaging revealed a thoracic aorta pseudoaneurysm in the region of the ligamentum arteriosum (Figure 1) and a duodenal transection (Figure 2).

We decided to first treat the duodenal trauma with an exploratory laparotomy. Prior to the start of the operation we discussed the aortic injury with anesthesia and emphasized the need for heart rate and blood pressure control. The patient was placed on an OR table compatible for fluoroscopy to also allow for endovascular intervention in the same setting. During the exploration, we noted a grade 3 duodenal injury involving 60% of the circumference of the second portion of the duodenum. The ampulla was visualized and was not involved. The decision was then made to close the injury by primary repair in two layers including inner 2-0 Vicryl sutures in a continuous manner and a second layer of Lembert stitches using 2-0 silk. Pyloric exclusion was considered, but given the feasibility of the primary repair we opted not to exclude. An omental patch was secured in overlay over the repair. A distal jejunostomy was placed for feeding and three large JP drains were placed posterior, anterior, and lateral to the repair. A nasogastric tube was kept in place. At the completion of the duodenal repair, the patient remained hemodynamically normal and demonstrated no signs of hypothermia or instability. The patient seemed physiologically appropriate to tolerate a second procedure. At this point, a joint team of vascular and cardiothoracic surgery proceeded to repair the thoracic aorta in the same setting.

An endovascular approach to his aorta was taken by right common femoral artery cut-down. An arch aortogram was initially performed showing evidence of a bovine arch. The site of aortic transection was identified distal to the left subclavian artery with an associated pseudoaneurysm. There was no active extravasation of contrast. An endovascular stent was used to cover the transection site and left subclavian artery and terminated at the bovine insertion of left common carotid artery proximally to obtain adequate seal. A 28 mm × 120 mm thoracic endovascular prosthesis was deployed for the repair. Repeat aortogram revealed complete coverage without evidence of leak and good flow through bovine arch. The left subclavian remained perfused through collaterals. The arteriotomy and the groin wound were closed and the patient remained intubated at the end of the case.

The patient was brought to the surgical ICU, where he was extubated and transferred to the floor the following day. J-tube feeds were started on postoperative day (POD) 2. The patient also had his right humerus fracture repaired on POD 4. He underwent an upper GI series with gastrografin on POD 6 that demonstrated no leak. His NGT tube and drains were subsequently removed, and he was started on oral diet. He was discharged home POD 8 with the j-tube in place. A CT scan obtained prior to discharge showed the endovascular stent to be in good position.

## 3. Discussion

The management of both duodenal and traumatic thoracic injuries has evolved over the past few decades. Duodenal injuries are classified according to the American Association Society of Trauma (AAST) organ injury scale [3]. This patient has a grade 3 duodenal injury involving 60% of the circumference of the second portion of the duodenum. Care has to be taken to inspect whether the ampulla is involved in the injury. Various techniques for duodenal injury repair have been developed over the years. Techniques such as duodenal diverticulization, “triple tube therapy” and pyloric exclusion have been described. The duodenal diverticulization and triple tube therapy approach have been abandoned due to technical complexity and susceptibility to complications [4]. Pyloric exclusion and gastrojejunostomy was initially described in 1909 and modified in the early 1980s as a technique to divert enteric inflow past the injury and allow healing while preventing duodenal fistula. Controversy remains over the optimal treatment method due the rarity of these injuries [5]. More recent studies have not demonstrated benefit to this approach over primary repair. Primary repair when feasible has been shown to have lower morbidity [6]. In very rare cases, often destructive injuries involving the Ampulla, a pancreatoduodenectomy, may be necessary.

With the evolution of endovascular techniques, repair of the thoracic aorta by endovascular approach has become the method of choice to repair these injuries [7]. Endovascular repair is associated with less morbidity and mortality. The use of endovascular repairs has decreased overall mortality from 22% to 13% and procedure related paraplegia from 8.7% to 1.6% [8]. Medical management of blunt aortic injury includes heart rate and blood pressure control until definitive repair [9]. The time interval to definitive repair has increased with increased use of Thoracic Endovascular Aortic Repair (TEVAR) compared to early repair with open approaches [8]. This also permits delayed repairs in patients who require a damage control approach for concomitant traumatic injuries. A recent multicenter review demonstrated the shift in management approach over the past decade [8]. More patients are undergoing an endovascular repair versus open with less morbidity and mortality with larger interval delays to definitive treatment. However long term data regarding durability is unavailable. In this case the patient was hemodynamically stable after the exploratory laparotomy and the aorta was repaired in the same setting.

## 4. Conclusion

Duodenal and blunt thoracic aorta injures have been traditionally associated with a high degree with morbidity and mortality. With evolution of repair techniques for both injuries patients can be expected to survive these injuries with less morbidity and faster recoveries.
